# Learned response dynamics reflect stimulus timing and encode temporal expectation violations in superficial layers of mouse V1

**DOI:** 10.1101/2024.01.20.576433

**Published:** 2024-01-22

**Authors:** Scott G. Knudstrup, Catalina Martinez Reyes, Jeffrey P. Gavornik

**Affiliations:** 1 Center for Systems Neuroscience, Department of Biology, Boston University, Boston, MA 02215.; 2 Neurophotonics Center, Boston University, Boston, MA, 02215.; 3 Graduate Program in Neuroscience, Boston University, Boston, MA 02215.

## Abstract

The ability to recognize ordered event sequences is a fundamental component of sensory cognition and underlies the capacity to generate temporally specific expectations of future events based on previous experience. Various lines of evidence suggest that the primary visual cortex participates in some form of predictive processing, but many details remain ambiguous. Here we use two-photon calcium imaging in layer 2/3 of the mouse primary visual cortex (V1) to study changes to neural activity under a multi-day sequence learning paradigm with respect to prediction error responses, stimulus encoding, and time. We find increased neural activity at the time an expected, but omitted, stimulus would have occurred but no significant prediction error responses following an unexpected stimulus substitution. Sequence representations became sparser and less correlated with training, although these changes had no effect on decoding accuracy of stimulus identity or timing. Additionally, we find that experience modifies the temporal structure of stimulus responses to produce a bias towards predictive stimulus-locked activity. Finally, we find significant temporal structure during intersequence rest periods that was largely unchanged by training.

## Introduction

Repeated exposure to visual sequences shapes responses in mouse primary visual cortex (V1) in a sequence- and timing-specific manner (Gavornik & Bear, 2014; Price et al., 2023; Sidorov et al., 2020; Tang et al., 2023). While the functional implication of these modifications is unclear, proponents of predictive processing theories have posited that the changes in visually evoked responses over days of spatiotemporal sequence exposure represent a physiological consequence of plasticity through which cortical circuits learn to predict statistical regularities in the environment. According to the predictive coding model (recently reviewed in Keller & Mrsic-Flogel, 2018), heightened responses when visual inputs do not match internally generated predictions constitute a prediction-error signal that carries information about the identity of the unexpected stimulus and the degree of its unlikelihoodClick or tap here to enter text.. While a variety of evidence from primary sensory regions supports this basic framework (Audette & Schneider, 2023; Eliades & Wang, 2008; Fiser et al., 2016; Keller et al., 2012; Stanley & Miall, 2007; Zmarz & Keller, 2016), the degree to which predictive coding theories can explain visually evoked responses is unclear. Our lab recently used extracellularly recorded multi-unit activity to investigate how passive exposure to spatiotemporal patterns modifies cells at the layer 4/5 boundary (Price et al., 2023) and found evidence for temporally specific activity consistent with prediction errors in these cells though not to the extent originally observed with LFP-based recordings (Gavornik & Bear, 2014). The convergence of bottom-up and top-down signals in L2/3 suggests that it hosts comparison circuits required in the predictive coding model, an idea supported by recent experimental in context of visuomotor feedback (Jordan & Keller, 2020). Accordingly, we sought to determine whether cells in L2/3 generate prediction errors when presented with modifications to a training sequence repeatedly viewed over 5 days of training.

One notable aspect of our previous work is that responses to a predicted sequence are modified when the constituent elements are presented with the expected order but novel timing. Accurately predicting when events will occur requires forming memories that explicitly encode temporal durations and some sort of internal clock to track elapsed time relative to stimulus events. While temporal processing has been studied in many brain areas, including hippocampus (Kraus et al., 2013; MacDonald et al., 2011; Pastalkova et al., 2008), entorhinal cortex (Heys & Dombeck, 2018; Kraus et al., 2015; Tsao et al., 2018), sensory thalamus (Komura et al., 2001), and motor cortex (Balasubramaniam et al., 2021) and the visual cortex (Benucci et al., 2009; Price et al., 2023), there are still gaps in understanding how experience encodes temporal expectations into neural circuits.

We used an implicit sequence learning paradigm and two-photon Ca imaging to study how exposure to visual sequences shape L2/3 responses in head-fixed mice when ordinal or temporal expectations are violated. After baseline imaging sessions, mice were exposed to a single training sequence for four consecutive days. On the fifth day, we exposed the mice to the training sequence and two test sequences designed to elicit temporal or ordinal prediction errors. We found little evidence of prediction errors when elements were presented with an unexpected order, but did find elevated activity at the time an omitted element should have been presented that we interpret as a form of temporal prediction error. Though evoked response dynamics are modified with training, decorrelating and shifting towards stimulus-locked responses, this has no obvious advantage for decoding elapsed time or stimulus identity at the population level. Finally, we find temporally specific activity in the gray inter-sequence period.

## Results

### Experimental design

**Figure 1:**
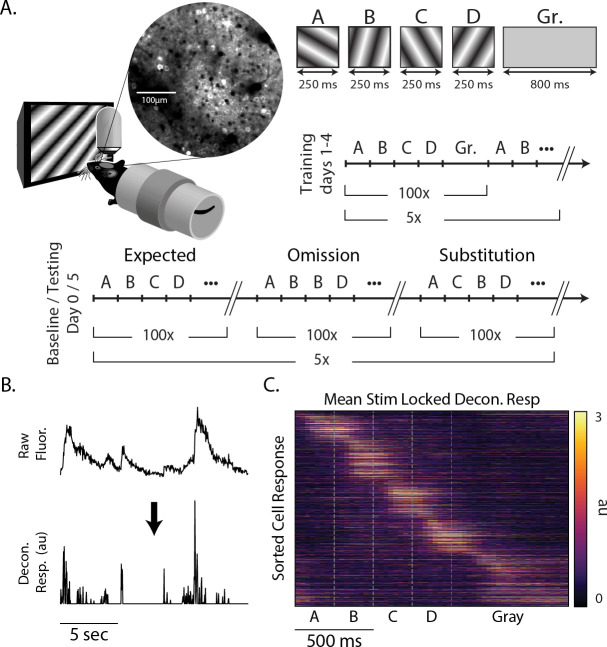
Experimental design (A) Awake head-fixed mice viewed sequences of oriented gratings while undergoing two-photon imaging of V1. Location of V1 was determined by widefield retinotopic mapping prior to experiment. Mice saw ABCD, ABBD, and ACBD for day 0 (pre-training) and day 5 (test) and ABCD only for the four days in between (training). Each image was shown for 250 ms, and sequences were separated by an 800 ms gray screen. (B) Fluorescence extracted from ROIs was deconvolved prior to analysis. (C) Trial-averaged responses of 1368 cells on day 0 to sequence ABCD.

We used a multiday experimental protocol in which mice were exposed to a standard sequence (ABCD) and two variants (ABBD and ACBD) designed to elicit positive (unexpected element C in the B position) and negative (element C omitted by keeping B on the screen for twice the expected duration) prediction errors (Figure 1A). To establish a baseline for comparison, mice viewed all three sequences on day 0 (pre-training). After two days with no visual stimulation, mice were then show the ABCD sequence exclusively during days 1–4 (training). On day 5, mice saw all three sequences again (testing). The training period serves to build an expectation for the sequence ABCD, while the day 0 baseline allowed us to compare responses to all sequences before and after the ABCD had been established as the expected sequence.

Mice were awake and head fixed while viewing stimuli during all sessions. Sequences were composed of four oriented gratings, each of which was presented for 250 ms for a total sequence length of 1 second and sequence presentations were separated by 800 ms of gray screen. Sequences were presented in 5 blocks of 100 with a 30 second gray period between blocks. Since gratings transitioned directly one into another, element B in ABBD was essentially a single element lasting 500 ms and there was no visual indicator of when the first B ended and second begam. We notate individual sequence elements using bold lettering (e.g., A**B**CD refers to B in ABCD). As detailed in the Methods section, ROIs designating visually responsive somas were identified. The average pixel fluorescence value within each ROI was calculated for each frame and this signal was deconvolved in time (Pachitariu et al., 2016) to produce an activity metric with a relatively high degree of temporal precision relative to the underlying calcium signal (figure 1B). This approach produced a population of neural responses that clearly shows unique responses to each element of the sequence with activity spanning the entire period of stimulation (figure 1C).

Binocular visual cortex was identified via retinotopic stimulation (see methods) and landmarks from reference images taken on day 0 were used to target same approximate population of neurons on 5. We imaged a total of 1368 and 1500 cells on days 0 and 1, respectively from 8 mice. Cells were categorized by stimulus-selectivity and visual responsiveness within the sequence (see Table 1). We did not track the response properties of individual neurons across training days, but the percent of neurons representing each element decreased by an average of 2.8 % between days 0 and 1 while the number of cells classified as gray responsive stayed approximately the same.

### Omissions, but not substitutions, drive prediction errors

**Figure 2:**
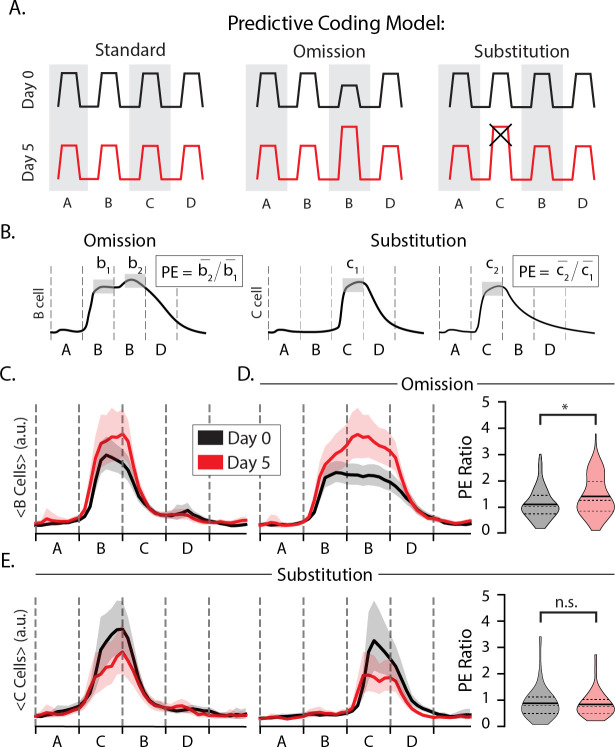
Prediction errors (A) Diagram of putative prediction error (PE) responses to omissions (middle) and substitutions (right) where the omitted/substituted element is expected to drive elevated responses on day 5 compared with day 0 (x indicates that this response is not present in our data). (B) PE ratios were computed by dividing trial- and time-averaged activity to the deviant image by activity during a corresponding standard image. (C) Average trace of B-responsive cells to ABCD with bootstrapped 95% confidence intervals. (D) Average trace of B-responsive cells to ABBD (left) and distributions of omission-type PE ratios (right) on days 0 (gray) and 5 (red). Note that there is no change in visual stimulus at the B_1_B_2_ transition. On day 0, mean PE=1.1 (n=138). On day 5, mean PE=1.4 (n=107). Distributions were significantly different (p << 0.05; KS-test). (E) Average trace of C-responsive cells to ACBD (left) and ABCD (middle). (right) Distributions of substitution-type PE ratios for C-responsive cells. On day 0, mean PE=0.88 (n=88). On day 5, mean PE=0.84 (n=39). Day 0 and day 5 distributions were not significantly different (p>0.05; KS-test).

The first goal of our experiments was to test whether layer 2/3 neurons display prediction errors in response to stimulus omissions or substitutions. The sequence ABBD contains an omission violation in sequence position 3 where since B is held on screen. The sequence ACBD contains a substitution violation at the second element since C appears where B is expected. The predictive coding model holds that both forms of expectation violation should result in elevated responses on day 5 relative to the day 0 baseline as diagramed in figure 2A.

We first classified cells based on their stimulus-selectivity with and without regard for sequence context (see Methods). This approach was designed to filter for cells that, for example, fire preferentially to element C in at either ABCD or ACBD. In this example, a cell that fires more during A**C**BD than AB**C**D would exhibit a pattern of excessive activity associated with prediction errors signals. We quantified this effect using a prediction error metric (PE) where the trial- and time-averaged activity during an unexpected event is divided by the activity during a similar but expected event. If training drives changes to prediction error responses, then the distribution of PE ratios should be significantly different before and after training.

To look for omission errors, we filtered for B-responsive cells and computed their PE ratios using AB**B**D and A**B**BD as the unexpected and expected stimuli, respectively (figure 2B, left). The average response to element B in position 2 increased slightly with training (figure 2C) and was approximately the same during the second position regardless of which stimulus followed on days 0 and 5 (figure 2 C,D). Training produced significant differences in the temporal characteristics of B-responsive cells. On day 0, activity in B responsive neurons decreased consistently as the B element was held into position 3. After training, however, activity was elevated during this period with a slight increase following the point in time at which element C would normally be seen (figure 2D). The PE ratio increased significantly with training (p = 0.005, n=276; KS-test). We interpret this result, wherein evoked activity increases at a point in time where visual inputs are unchanging, as representing a temporally specific prediction error following the omission of an expected visual transition. This finding is broadly consistent with our previous findings in deep layer 4 (Price et al., 2023) and suggest that temporal prediction errors can be found in the population of excitatory neurons selective for an expected stimulus. We did not identify a separate population of otherwise sequence non-responsive “prediction error cells” uniquely activated during stimulus omissions as posited by some predictive coding models.

To look for substitution errors, we filtered for C-responsive cells and computed their PE ratios using the C response in both the unexpected and expected position within the sequence (figure 2B, right). If substitution drives a prediction error in following an unexpected substitution, the PE ratios for these cells would be approximately equal to 1 at baseline and higher than 1 after training. The average response to element C was higher at baseline than on day 5 (figure 2E). We found that mean PE ratios were approximately equal to 1 on both days, with a slight but statistically insignificant decrease following training (p = 0.64, n = 160; KS-test). Contrary to our expectations coming into this experiment, training did not facilitate a significant change in responses to the expected vs unexpected element during element substitutions. As with during the omission case, we did not identify a unique population of otherwise visually non-responsive “prediction error cells” following substitution. Overall, and in contrast to our previous publications, we find no evidence that unexpected ordinal substitutions drive elevated activity consistent with predictive coding models in excitatory layer 2/3 cells.

### Experience simplifies activity in principal component space and drives sparsification

**Figure 3:**
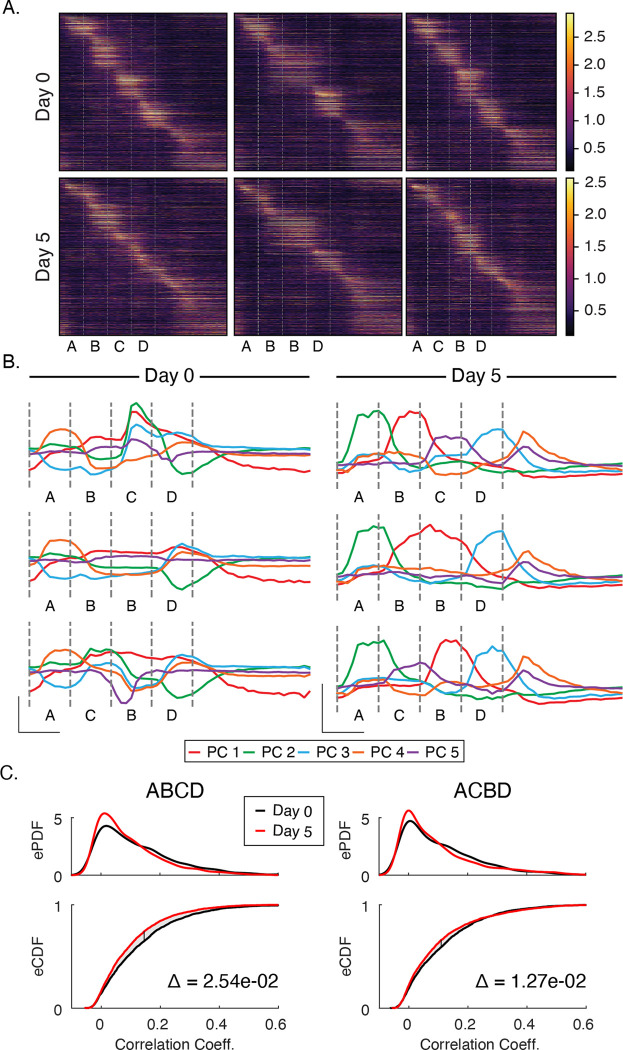
Principal Component and Sparsity Analysis (A) Trial average population responses, sorted by time to peak latency in each cell, to each sequence before and after training. (B) Prior to training, activity is driven along principal components jointly in complex combinations. After training, each of the most significant principal components correspond neatly to individual stimuli. In both datasets, the first five components explain ~80% of the variance. (C) To test whether changes in principal component space reflected the decorrelation of responses, we computed Pearson-correlation coefficients between all four images for each sequence presentation individually. Empirical PDFs (top panels) and CDFs (bottom panels) of Pearson-correlation coefficients. After training, activity became significantly less correlated (p < 0.05; KS-test) for ABCD and ACBD. Delta (Δ) on bottom panels indicates area between curves on CDFs.

Sequence responses on day 5 look qualitatively different at the population level relative to baseline (figure 3A). To quantify this observation, we performed principal component analysis on pre- and post-training datasets. Prior to training, stimuli drive activity along several axes in principal component space in complex combinations and the principal components do neat neatly reflect activity driven by any particular sequence element (figure 3B). In contrast, activity in principal component space is highly discretized or “untangled” after training with clearly defined peaks reflecting sequence elements. Though the total number of principle components required to account for 90% of total variance did not change appreciably with training (approximately 10 PCs on days 0 and 5), the components accounting for the majority of variance neatly reflect specific sequence elements after training.

Training also facilitated a reduction in the fraction of neurons that responded preferentially to individual stimuli. Prior to training, 40.8% of neurons were driven more than two standard deviations above their baseline firing rate by one of the stimuli compared with 27.1% after training. We also quantified how many cells were “visually modulated”, a more permissive metric that simply compares activity between gray and non-gray periods. Under this measure, the percentages of visually modulated cells went from 72% on day 0 to 60% after training. Overall, responses are sparser after training than at baseline.

Since information theory holds that codes gain efficiency by eliminating redundant information (see Price & Gavornik 2022 of how this relates to predictive coding in the visual system), we also examined correlation coefficients between stimuli to determine if our day 5 activity was less correlated than day 0 activity. For each sequence presentation, we calculated the Pearson correlation coefficients between all pairs of stimuli, yielding a collection of coefficients for each sequence on each day (Figure 3C). We found that correlations were reduced by 10–20% on day 5 compared with day 0. Across all sequences, the differences in correlation distributions were significantly different between days (ABCD: p < 1e-3, n=6000; ACBD: p < 1e-3, n=6000; KS-test). This, coupled with the overall decrease in number of cells responding to visual stimulation, suggests that experience-dependent spatiotemporal plasticity increases coding efficiency.

### Plasticity does not increase decoding accuracy

**Figure 4:**
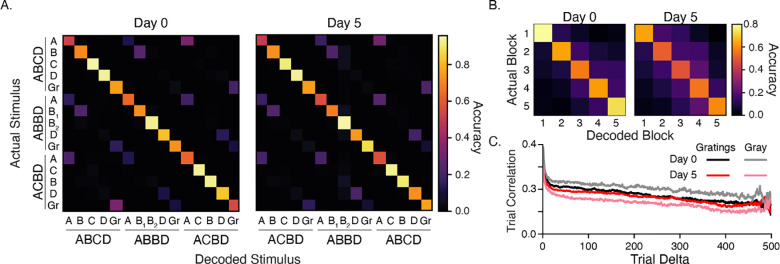
Stimulus responses (A) Heatmaps display trial-averaged responses to all sequences sorted by peak response to ABCD. Histograms reflect locations of peak activity for each cell and shift towards stimulus locked phasic responses after training. Red and green rectangles highlight area of decreased and increased activity on day 5 relative to baseline. (B) Combining histograms across all stimulus conditions shows how temporal response latency patterning changes over days. Note that the first two time bins (66 ms) after onsets are omitted in histograms to compensate for the transmission delay from retina to L2/3 cells. Prior to training, responses slowly build up after onset. After training, responses are robust at onset and undergo quick depression prior to ramping up for the next element. Histograms based only on early trials (first 100) show that this pattern does not change significantly over the course of the imaging session. (C) To test whether changes to temporal patterning might reflect a change in the ability to discern durations, we trained decoders on responses from different time bins following sequence onset. Decoder accuracy did not change significantly with training.

Our principal component analysis suggests that experience creates representations that are more easily separable in high-dimensional space. To test whether experiential shaping of cortical circuits increases the ability to differentiate between different visual stimuli, we trained a linear decoder with responses from all stimulus contexts (Figure 4A). We found that accuracy was well above chance (1/15 = 6.7% ± 0.4%) on day 0 (78%) and day 5 (76%) and that the decoder was able to discriminate between cases we expected to be indistinguishable. For example, we expected **A**BCD would be more-or-less indistinguishable from **A**BBD and **A**CBD since A occurs first in each sequence and always preceded by a long (800 ms) gray period. This was not the case. Most often, the decoder correctly identified which sequence stimulus A came from. This pattern was observed with other cases as well, including the intersequence gray periods.

We next trained a decoder with data from the same sequence element (e.g., **A**BCD) and found that decoder accurately predicted which block the stimulus came from and that the rate of false positives decreased with time (Figure 4B). This implied a slow, continuous change in stimulus representations. We found no significant change in overall firing rates that might indicate that this effect could be a consequence of adaptation, repetition suppression, or photobleaching (effects that should be minimized anyway by our use of deconvolved data).

To understand how the decoder was able to accurately determine which block stimuli came from, we computed Pearson-correlation coefficients between all population vectors for a given sequence element and found that vector similarity falls away with time between presentations (Figure 4C). These findings suggest that there is a measurable amount of representational drift across a single 30 min recording session in V1 and are consistent with a previous work focused representational on drift in V1 (Deitch et al., 2021). The amount of drift within a session was comparable on days 0 and 5, with a slightly elevated drift rate on day 5. This was especially pronounced in gray periods, which were significantly more variable on day 5.

### Experience facilitates stimulus-locked responses and temporal echoes

**Figure 5:**
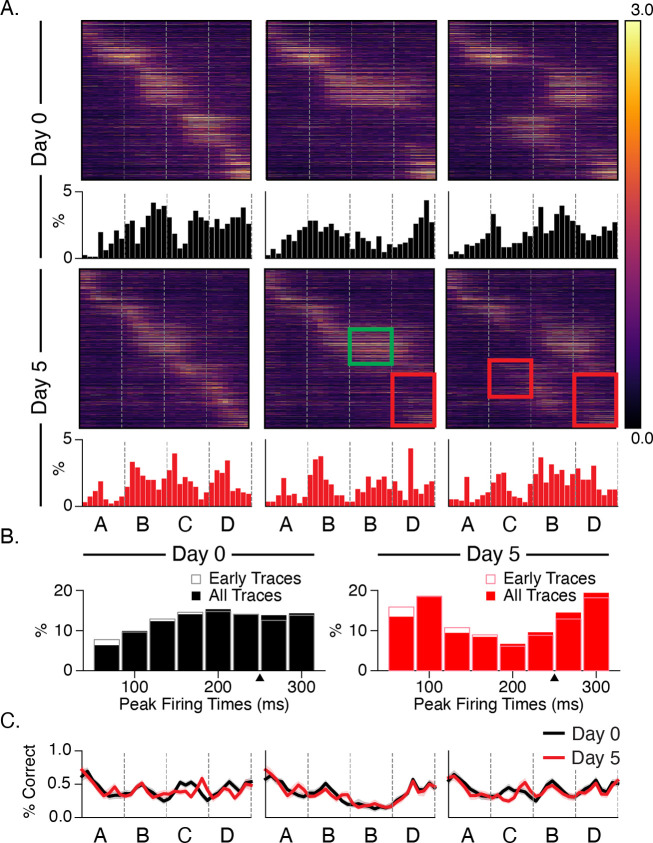
Gray responses (A) Heatmaps displaying trial-averaged responses during gray periods following sequence presentations. Cells were sorted by peak response time to ABCD (top panels) and gray periods only (middle panels). Histograms (bottom) reflect locations of peak activity for each cell and show that there is an increase in active population size about 300 ms after gray onset and a second uptick towards preceding the next sequence presentation at 800 ms. (B) Average confusion matrices (100 iterations) for decoders trained on responses at different delays from gray onset for day 0 (left), day 5 (middle), and the difference between them (right). (C) Overall decoder accuracy as a function of time since gray onset did not change significantly with training.

To analyze changes in stimulus locking and how post-onset durations are represented in the brain, we assigned a time bin to each cell based on its point of maximal activity (Figure 5A, also see Methods). At baseline, few cells have peak firing times immediately after stimulus onset and the percent of cells with late maximal response times increases gradually over each stimulus (Figure 5B). After training, the pattern is reversed. More cells fire maximally at or near stimulus onset, and the number of cells that fire at intermediate time points decreases, and there is a sizeable population of cells that fires immediately before the next stimulus transition. There is no obvious shift in response latencies following stimulus substitution or omission, though the response profile to element D is disrupted in both cases after training relative to baseline. Despite the shift in temporal response latency profiles, the ability of a linear decoder to determine time within the stimulation period did not increase following training (Figure 5C).

**Figure 6:**
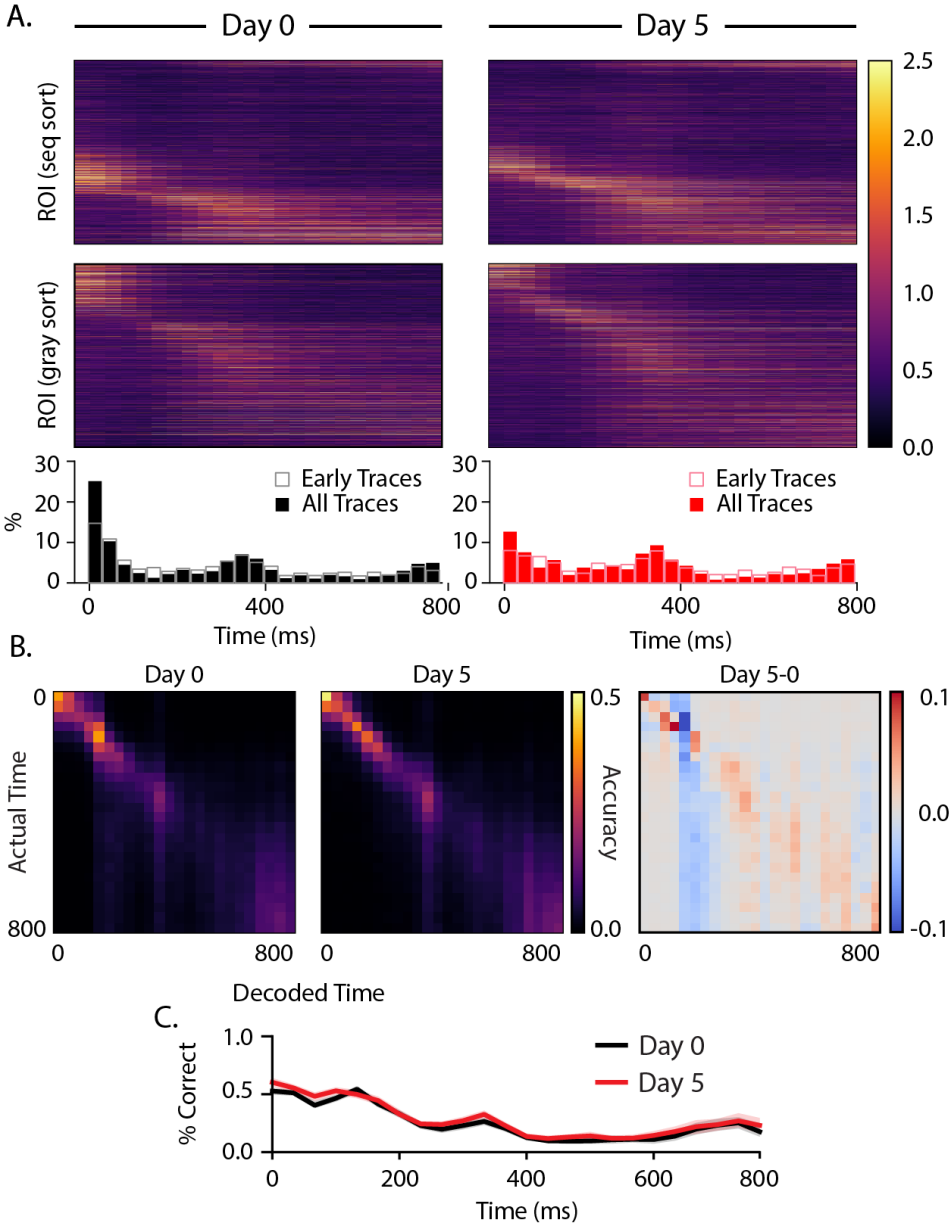
Stimulus decoding and representational drift (A) Average confusion matrices (100 iterations) for decoders trained on responses to all images. Average decoder accuracy was 78% and 76% for days 0 and 5, respectively. Both are well above chance (6.7% ± 0.4%). The ability to differentiate correctly between the same image in different contexts, such as ABCD vs ABBD, prompted us to consider whether responses slowly drifted over time since sequences were presented in large blocks. (B) A decoder trained on individual elements (for example, ABCD) accurately classifies which block responses came from, with errors decreasing along with distance between blocks (e.g., block 1 is often confused with block 2 but not block 5). Decoder accuracy was 68% on day 0 and 56% on day 5. (C) We measured drift by computing Pearson-correlation coefficients between all pairs of population vectors driven by a particular sequence element and grouped these values by how far apart the pairs were in time/trial. Responses clearly become less correlated as distance between trials increases during both stimulus-evoked and gray periods. The largest change in overall temporal correlation was seen between gray periods on days 0 and 5.

We performed the same analysis on the intersequence gray periods and found that a subpopulation of cells was activated ~300 ms after gray period onset (Figure 6), slightly later than the 250 ms interval suggested by the temporal structure of our visual sequence. A second population of cells exhibited ramping activity leading up to the end of the 800 ms gray period. While this response looks like it anticipates the onset of the next sequence, it is present at baseline including in early trials and did not change significantly with training. The only noticeable difference after training is a decrease in the percentage of cells firing early in the gray period on day 5 relative to baseline. Overall, the static nature of temporal firing patterns during the gray period contrasts with the evolution of stimulus-driven temporal patterning over days. Temporal decoding accuracy during the gray period was highest early, with small increases around the 300 ms peak and 800 ms ramp. As we saw with sequence evoked responses, temporal decoding accuracy did not change significantly over days.

To assess whether these changes in temporal pattern allowed the brain to more precisely represent durations, we performed trained a decoder on population vectors classified by their time bin and asked the decoder to classified testing data which time bin it belonged to (Figures 5C, 6C). Decoding was performed 100 times from which mean and 95% confidence intervals for decoder accuracy were generated. We found no significant change in temporal decoding during either sequence or intersequence gray periods. On average, decoding accuracy was 23.4% on day 0 and 23.4% on day 5. Both are well above chance (3.1% ± 0.2%). However, we did notice that decoding accuracy does not decline uniformly after the onset of a gray period. There are moderate increases in decoding accuracy in about 300–400 ms and nearing the end of the 800 ms gray period.

## Discussion

In this study, we used chronic two-photon imaging to measure activity in L2/3 of mouse V1 during passive exposure to standard and deviant visual sequences before and after a four-day training period. This study was designed to test the hypotheses that neurons in L2/3 exhibit prediction error responses in the form of elevated activity elicited expectation violations by comparing how V1 responded to identical sequence before and after exposure established an expectation of how different sequence elements relate to each other in time. We focused on two types of violations (omissions and substitutions) and found mixed support for hypothesized prediction errors. We also found that experience yielded a sparsification of neural responses in the form of fewer visually responsive cells. Response vectors in principal component space were also altered by experience, yielding simpler more compartmentalized representations after training. Finally, we found changes in the temporal code that reveal a reorganization of stimulus-driven activity and possible evidence of temporal predictions.

When an expected transition was omitted by holding the preceding image on the screen, we found an elevated response after training that peaked when the omitted element would have been seen and was sustained throughout the omitted stimulus period. This contrasted with a decaying response observed at baseline. The temporal specificity of the response supports our previous findings that V1 responses are actively modulated by temporal expectations formed during exposure to spatiotemporal visual sequences (Price et al., 2023). Other work outside of our lab has provided evidence for omission-errors in L2/3 of V1, but the experimental paradigms vary in ways that make direct comparisons difficult. Fiser et al. 2016 found a subset of cells that responded anticipatorily to an omitted grating that was expected be found at a particular location along a linear track. In this case, the task involved spatial location, locomotion, water reward, and perhaps most crucially, the complete omission of a grating at an expected location rather than a held-over grating and no predetermined timing of the grating presentations. In another study, Garrett *et al.* observed ramping activity in vasoactive intestinal peptide (VIP) expressing inhibitory cells but not excitatory cells in L2/3 in response to an omitted image (Garrett et al., 2020). The images were separated by 500 ms gray periods and omitted activity could have been predicated in part by visual transitions that were absent here and in Price et al. 2023.

When an expected grating was substituted with a different (but familiar) grating, we did not observe elevated activity consistent with predictive coding theory. As with omission-type errors, we implemented a strategy to filter out cells deemed non-responsive to the substituted element in any context since 1. predictive coding theory posits that prediction-error signals are feature-specific (Keller & Mrsic-Flogel, 2018) and 2. such a highly specific signal may be too small in comparison with total activity to elicit noticeable changes in global activity levels. This negative result is consistent with recent work in monkey V1 and human EEG that found little evidence of substitution-type prediction errors in a conceptually similar experiment involving passive exposure to standard and deviant visual sequences (Solomon et al., 2021). However, in Solomon et al., the electrodes read from an unknown cortical depth, and so it is possible that few prediction error responses were found due to a mismatch between lamina recorded and lamina involved in prediction errors. It is also possible that the dynamics of calcium imaging (both the dynamics of the calcium signals and frame rate of two photon acquisition) are too slow to accurately capture prediction error responses. This has been suggested as an explanation for why Stimulus Selective Response potentiation, a similar but mechanistically distinct form of visual plasticity, is not readily apparent in calcium signals (Montgomery et al., 2022). Recent work in the auditory cortex found that prediction error cells respond with rapid, transient responses lasting only about 40 ms (Audette and Schneider, 2023). Finally, we cannot rule out that exposure to the ACBD sequence during baseline prevented an error by durably encoding this sequence as “familiar”. Regardless, this finding is in relatively stark contrast to the large effect seen in LFP data when sequence elements are reordered after training (Gavornik & Bear, 2014; Sidorov et al., 2020). This discrepancy could reflect the fact that the LFP represents dendritic currents (e.g. inputs, see Buzsáki et al. 2012) rather than somatic spiking (e.g. outputs) and includes inhibitory activity that we are functionally blind to in these experiments. Additional work is required to resolve this issue.

Consistent with principles of efficient coding (Price & Gavornik 2022), we found that training had the effect of creating relatively sparse response patterns. Using two different measures, we estimate that the training reduced the number of visually modulated cells by 20–30%. The sparsification may be related with the qualitative changes in principal component space where training creates principal components with dynamics that neatly corresponded to individual stimuli. In addition to being more efficient, a sparser orthogonalized code could also be easier to other brain regions to interpret. Admittedly, this argument would be stronger if element or temporal decoding accuracy had increased with training. While it is certainly true that brain processes operate very differently than a linear decoder, and may take greater advantage of modified dynamics, it is also worth noting that the decoders on day 5 were as accurate as those trained against baseline data despite the smaller proportion of visually responsive neurons on day 5.

Neural sequences have been proposed as one of several mechanisms for representing durations, and there is growing support for this regime in different parts of the brain (Tsao et al., 2022). In this view, different cells fire preferentially at different delays relative to the onset of an external event, thereby forming a stable neural trajectory from which different points in time can be read out. We speculated that the distribution of observed “time fields” (i.e., locations of peak activity) might be altered with training in some way to reflect the 250 ms stimulus durations. By focusing our analysis on cells with consistently timed activity, we found significant differences in sequential activation following training. Prior to training, relatively few cells peaked at or near stimulus onset, and time fields distributions became increasingly densely throughout the ~200 ms post-onset window (Figure 5B). After training, more cells peaked at stimulus onsets, and intermediate durations were less densely represented. Our analysis of time fields during the interstimulus gray period showed a surprising degree of temporal structure, though no evidence of experience dependent plasticity. A cluster of peaks in the 300 and 750 ms ranges are tantalizingly close to the 250 ms element time and 800 ms gray period intervals, but the fact that they are in early traces from baseline recordings makes it unlikely to reflect anything like a learned response. These peaks might be related to visually evoked theta-range oscillations reported previously (Gao et al., 2021; Kissinger et al., 2020; Levy et al., 2017; Zold & Hussain Shuler, 2015) though these are reported to develop with familiarity (Kissinger et al., 2018).

Our findings build on previous work to show that sequence plasticity modifies evoked responses in superficial layers in a manner consistent with that seen in thalamocortical input layers. Overall, the work continues the recent tradition of providing ambiguous support for the idea that cortical dynamics are best described by predictive coding models while simultaneously demonstrating that evoked dynamics in V1 are far more complex than canonical visual processing models suggest.

## Methods

### Animal subjects

A total of eight mice (3 male, 5 female) aged 2–5 months were used for this study [CaMKII-tTA:tetO-GCaMP6s (Jackson Laboratories stock numbers 007004 and 024742)]. Mice were housed in a climate-controlled environment on a standard 12-hour light-dark cycle and were provided with food and water *ad libitum*. Cranial windows were implanted in mice at P40-P70. Experiments were performed during the mouse’s light cycle. All procedures were approved by the Institutional Animal Care and Use Committee (IACUC) of Boston University.

### Cranial Windows

Mice were briefly anesthetized with isoflurane (~2% by volume in O2) and placed on a stereotactic surgical stage with warming pads to maintain body temperature. Anesthetic gas (1–2%) was passively applied through a nose mask and adjusted as necessary to maintain respiratory rate and suppressed hind-paw and tail-pinch reflexes. Eyes were coated with a thin layer of Soothe eye lubricant (Bausch and Lomb, Canada). The scalp was shaved and opened with a rostrocaudal incision at the midline using scissors, and the periosteum was removed. A 5 mm circular craniotomy was made over the left visual cortex (2.9 mm lateral and 0.5 mm anterior to lambda). A 5 mm cranial window (Deckglaser) was placed over the craniotomy and secured with metabond. A steel headplate was then affixed to the skull. Mice recovered for 7–10 days prior to imaging.

### Retinotopic Mapping

1–3 days prior to beginning the sequence learning experiment, a retinotopic map of the visual cortex was generated using widefield one-photon microscopy. The brain was illuminated with a 470 nm light source (X-Cite 200DC), and images were acquired through a 10x objective, a sCMOS camera (Thorlabs Quantalux, CS2100M-SB), and ThorImageLS 3.0 (Thorlabs Inc.). A 22-inch LED monitor (1920 × 1080 pixels, refresh rate 60 Hz) was positioned 15 cm from the mouse’s right eye, and a narrow sweeping stimulus moved across the screen in four directions (left-to-right, right-to-left, top-to-bottom, and bottom-to-top). Trial-averaged responses were used to determine the location of binocular V1.

### Two-photon Imaging

Two-photon Ca2+ imaging was performed on days 1 and 6 using a Bergamo microscope (Thorlabs Inc., Newton, NJ, USA) controlled by ThorImage OCT software (ThorImageLS, v3). The visual cortex was illuminated with a Ti:Sapphire fs-pulsed laser (Mai Tai Deep-See, Spectraphysics) tuned to 920 nm. The laser was focused onto L2/3 of binocular V1 through a 16x water-immersion objective lens (0.8NA, Nikon). Ca2+ transients were obtained from neuronal populations at a resolution of 512 × 512 pixels (sampling rate ~30 Hz). The obtained images were motion-corrected using CaImAn (Giovannucci et al., 2019). Segmentation, neuropil subtractions, and deconvolution was performed using Suite2p (Pachitariu et al., 2016).

### Visual Stimulus

Visual stimuli were generated and displayed using MATLAB with the PsychToolbox extension (Brainard, 1997), with custom software (https://github.com/jeffgavornik/VEPStimulusSuite) used to control timing and hardware signals. Stimuli were displayed on a 22-inch LED monitor (1920 × 1080 pixels, refresh rate 60 Hz) positioned 25 cm directly in front of the mouse in order to stimulate binocular V1. All stimuli were matched for luminance.

Four images (referred to A, B, C, and D), each an oriented sinusoidal grating, had angles 15, 75, 165, and 120 (respectively) with spatial frequency 0.05 cycles/degree. These images were displayed for 250 ms each. All images were matched for total luminance. Images were combined into three sequences (ABCD, ABBD, and ACBD). Every sequence presentation was followed by an 800 ms period of gray screen. Therefore, each sequence presentation, including its following gray period, lasted 1800 ms. Note: since there were no gray periods between images in a sequence, BB in ABBD was essentially a single 500 ms presentation of B.

### Experimental Design

On day 0, all sequences (ABCD, ABBD, and ACBD) were shown in blocks of 100 sequences (e.g. ABCD × 100, ABBD × 100, ACBD × 100), and each block was separated by a 10 second rest period (see Figure 1). This structure was repeated 5 times, yielding 500 presentations of each sequence on any given day. Day 0 and day 1 were separated by a two-day buffer period. During the training period (days 1–4), only ABCD was shown and in 5 blocks of 100 presentations. Day 5 (testing) was identical to day 0. Additionally, there was a 1-minute gray period preceding and post-ceding the experiment.

### Stimulus and context selectivity

Trial- and time-averaged responses were computed for all cells. We applied a 67 ms offset from stimulus onset to account for the information delay from retina to L2/3. The duration of this delay was verified by looking at individual cell responses after stimulus onset. For each cell, we compared the mean activity during for a given stimulus with the mean activity over all other stimuli. If the mean activity for a given stimulus was over two standard deviations of the other stimuli, it was assigned selectivity for that stimulus. This was first done for stimuli regardless of sequence context. The procedure was performed again with sequence context taken into account to look for cells that were primarily active within a particular sequence (e.g., cells that fired to image C in ACBD but not ABCD). Manual inspection of all cells (n=2868) validated this approach for >80% of cells. Some manual curation was performed to assign context selectivity when necessary.

### Principal Component Analysis

Trial-averaged responses for ABCD, ABBD, and ACBD were computed and pooled across mice. These responses were then concatenated across time yielding a time-by-cell matrix. After performing PCA, the resulting principal components were then split in time to show how the shared set of principal components behaved during each of the three sequences.

### Sparseness estimation

We estimated sparseness of single-cell responses in two ways.

1. Stimulus selectivity: We counted the number of neurons that are driven by a particular stimulus more than two standard deviations above their mean rates. For each neuron, we computed the trial- and time-averaged responses for each image. The mean firing rate and standard deviation was computed across time regardless of stimulus. A neuron was considered as responsive to a given stimulus if its response was more than two times this standard deviation plus its mean firing rate.
2. Visual modulation: We counted the number of cells that had significantly different activity during gratings vs gray periods. For each neuron, we collected and time averaged 266 ms chunks for gratings and gray periods separately. We then performed a ks-test on these two groups, and if the two distributions were significantly different (p < 0.05), then the cell was classified as visually modulated. Note that we threw away 133 mss in between 266 ms chunks in order to reduce correlations between samples.

### Correlation analysis

For each sequence presentation we extracted time-averaged responses for each stimulus (excluding the first 66 ms after image onset). We then computed Pearson-correlation coefficients for all 6 pairs in the presentation. For example, for the first presentation of ABCD, we would first extract and time-average population vectors for each image A, B, C, and D. We would then compute Pearson correlation coefficients between all 6 pairs: A to B, A to C, A to D, etc. In this way, we had 500 presentations × 6 pairs = 3000 coefficients for each sequence for each day. We compared coefficients between days with KS-tests.

### Stimulus decoding

Time-averaged responses to all stimuli were used to train a linear decoder. We excluded the first two time bins (67 ms) to account for the information delay to L2/3 and avoid contamination from the previous stimulus. Data was randomly split into two equally sized groups (250 trials each) for training and testing. This was repeated 100 times. Accuracy by random chance was 1/15 = 6.7%.

### Temporal decoding

Data from all 800 ms intersequence gray periods (n=1500) was collected and randomly split into two equally-sized groups (750 trials each). The decoder was trained on one set and tested on the other. This was repeated 100 times. Accuracy by random chance was 1/24 = 4.2%.

### Time field estimation

Trials were split into even/odd groups, and responses were averaged over trials for both groups. For each cell, we found where the cell fired maximally in each group. If the location of even-group maximum was within 133 ms (4 time bins) of the odd-group maximum, the cell was considered to have a temporally consistent firing pattern (~60% of cells on both days), and the remaining cells were discarded. Consistently firing cells were assigned a time bin based on their points of maximum activity using data averaged over all trials.

### Code/software

All data analysis was performed in Python using the Python scientific stack (Numpy, Scipy, Sci-Kit Learn). For decoder analysis, we used sci-kit learn’s support vector classifier with a linear kernel.

## Figures and Tables

**Table 1:** Stimulus selectivity

Stimulus	Day 0	Day 5	% change
A	84 (6.1 %)	41 (2.7%)	−3.4%
B	137 (10.0%)	106 (7.1%)	−2.9%
C	84 (6.1 %)	40 (2.7%)	−3.5%
D	126 (9.2%)	65 (4.3%)	−4.9%
Gray	129 (9.4%)	154 (10.3%)	0.8%

Stimulus-selectivity for day 0 (n=1368) and day 5 (n=1500). A cell was considered stimulus-selective for an element if the average activity evoked by that stimulus was more than two standard deviations higher than any other stimulus.

## Data Availability

All data and analysis code is available at https://gavorniklab.bu.edu/supplemental-materials.html.
